# The determinant of periodicity in *Mycoplasma pneumoniae* incidence: an insight from mathematical modelling

**DOI:** 10.1038/srep14473

**Published:** 2015-09-28

**Authors:** Ryosuke Omori, Yukihiko Nakata, Heidi L. Tessmer, Satowa Suzuki, Keigo Shibayama

**Affiliations:** 1 Division of Bioinformatics, Research Center for Zoonosis Control, Hokkaido University, Sapporo, 001-0020, Japan; 2 Graduate School of Mathematical Sciences, The University of Tokyo, Tokyo, 153-8914, Japan; 3 Department of Bacteriology II, National Institute of Infectious Diseases, Tokyo, 208-0011, Japan

## Abstract

Until the early 1990s, incidences of *Mycoplasma pneumoniae* (*MP*) infection showed three to five year epidemic cycles in multiple countries, however, the mechanism for the *MP* epidemic cycle has not been understood. Here, we investigate the determinant of periodicity in *MP* incidence by employing a mathematical model describing *MP* transmission dynamics. Three candidates for the determinant of periodicity were evaluated: school-term forcing, minor variance in the duration of immunity, and epidemiological interference between *MP* serotypes. We find that minor variation in the duration of immunity at the population level must be considered essential for the *MP* epidemic cycle because the *MP* cyclic incidence pattern did not replicate without it. Minor variation, in this case, is a less dispersed distribution for the duration of immunity than an exponential distribution. Various lengths of epidemic cycles, including cycles typically found in nature, e.g. three to five year cycles, were also observed when there was minor variance in the duration of immunity. The cyclic incidence pattern is robust even if there is epidemiological interference due to cross-immune protection, which is observed in the epidemiological data as negative correlation between epidemics per *MP* serotype.

*Mycoplasma pneumoniae* (*MP*) is a bacterium that causes bacterial pneumonia in humans and is a common cause of respiratory bacterial infection^1^. The most distinctive epidemiological feature of *MP* is its periodicity. In Denmark, the U.S., and Japan, three to five year cycles were observed up to the early 1990s^2^,^3^. Especially in Japan, the incidences of *MP* infection showed a clear four year epidemic cycle until the early 1990s, and so was commonly called “Olympic disease” among clinicians (Fig. 1a). These periodicities disappeared for reasons which are currently not well understood. A decline in *MP* incidence rates were observed with the widespread use of macrolides antibiotics^4^, but in recent years a macrolides-resistant strain of *MP* has emerged and macrolides-resistant *MP* cases have increased drastically^5^. With the drastic rise in macrolides-resistant *MP* cases, the need to be able to predict *MP* prevalence is growing. Understanding the dynamics of *MP* epidemics in the past is essential to predicting the prevalence in the future. However, the mechanism of oscillation in past *MP* epidemics has yet to be explained.

Many mechanisms of periodicity have been hypothesized to explain the oscillation of *MP* epidemics^6^,^7^,^8^,^9^,^10^,^11^,^12^,^13^. Among them is a seasonally-forced transmission rate. Most *MP* cases are observed among school children^14^ and also exhibit complex epidemic cycles as observed in other childhood diseases^15^,^16^. Mathematical models demonstrate that school-term forced transmission rates explain multi-annual dynamics in childhood diseases^12^,^15^,^16^. Other candidate mechanisms are the distribution of the infectious period, the distribution of the duration of immunity, and the latent period. Most mathematical models describing transmission dynamics of infectious diseases assume that the distribution of the sojourn time between host infection states follows an exponential distribution, however, conclusions and interpretations derived from mathematical modelling would be changed by using different assumptions for the distribution of the sojourn time^17^,^18^. Previous studies show that the dynamics become much more complex if the sojourn time follows a distribution pattern which is less dispersed than an exponential distribution^19^,^20^,^21^,^22^,^23^. Epidemiological interference is also a candidate mechanism of *MP* epidemic periodicity. *MP* is classified into two distinct serotypes with negative correlation between epidemics of different serotypes being observed (see Fig. 2a,b in Kenri *et al.* 2008^3^). This negative correlation suggests epidemiological interference between serotypes by cross-immune protection. Epidemiological interference itself does not induce sustained oscillation of the epidemic curve^24^, however, it affects the epidemic dynamics and can change the periodicity of epidemics triggered by other mechanisms^15^,^25^. As the effect of cross-immune reactions on the epidemic cycle of *MP* was not previously understood, we delve into the topic here.

In this paper, we assessed whether three mechanisms commonly used in mathematical models to describe the oscillation of the epidemic curve can, in fact, explain the periodicity of *MP* epidemics. These three mechanisms are: i) school term-forced transmission; ii) epidemiological interference by cross-immune reaction; and iii) minor variation in latent, infectious, and immunity periods. We began by analyzing whether i) and ii) are sufficient to explain *MP* epidemic periodicity within a simple SEIRS model. We then employed an SEIRS model with time delay for the assessment of a special case of iii), in which there is a constant duration for the latent, infectious, and immunity periods. Subsequently we assessed a more general case of iii) with individual-based Monte Carlo simulation.

## Results

### Epidemic periodicity when the sojourn time of each infectious state follows an exponential distribution

In this section, we assumed that the distribution of sojourn time of each infectious state follows an exponential distribution, and assessed the effect of cross-immune reaction and seasonal fluctuation of transmission rate on the oscillation of the epidemic curve using a deterministic model whose parameters are described in Table 1. If the transmission rate is constant over time and there is no cross-immune protection, there is no oscillation of the epidemic curve at equilibrium because endemic equilibrium is always stable, if it exists. Even if there is cross-immune protection, oscillation as seen in *MP* epidemics does not always exist if the transmission rate is constant over time^24^.

### School-term forcing

With respect to seasonality in the transmission rate, one possible explicit mechanism for the *MP* periodicity is the school term because most *MP* cases are observed among school children. The difference of behavior in school children, e.g. contact and movement between school terms and school holidays, is proposed as the cause of seasonality in the incidence of infectious diseases. The change in transmission rates between school terms and holidays is commonly referred to as ‘school-term forcing’^26^. Previous studies show that school-term forcing can explain the seasonality of measles epidemics well^16^.

So far, studies employing either SIR or SEIR models which take into account host birth and death rates reported that the periodicity of epidemics triggered by seasonally-fluctuating transmission rates is closely related to the basic reproductive number, *R*_0_, the length of the infectious period, and the amplitude of seasonally-fluctuating transmission rates^15^,^16^,^25^,^27^. Intermediate values of *R*_0_ are required for multi-annual epidemic cycles, while small or large *R*_0_ can induce only annual cycles^27^. Keeling *et al.* 2001^16^ reported that a short infectious period is required for sustained long-cycle oscillation of the epidemic curve. With respect to the amplitude of seasonally-fluctuating transmission rates, annual cycles are observed when the amplitude is small, and multi-annual cycles emerge when the amplitude is sufficiently large^15^,^25^.

We examined the effect of school-term forcing on *MP* epidemics. However, when parameters were set for *MP* as shown in Table 1 and the amplitude of school-term forcing *b* = 1.7 as the estimated *b* for measles in the U.K.^27^, the four year cycle was not observed. The results of previous studies are not comparable with our results due to the difference in model structure (with vs. without waning immunity), three possibilities can be considered for the reason why school-term forcing cannot explain the *MP* epidemic cycle; i) *R*_0_ of *MP* is out of range for multi-annual cycles, ii) the infectious period of *MP* is too long to induce multi-annual cycles, and iii) the amplitude of school-term forcing is not large enough to cause multi-annual cycles. With respect to *R*_0_, we explored the epidemic cycle using varying *R*_0_, from 1.5 to 20.5, however only annual cycles were observed. In terms of latent period and infectious period, we also explored the epidemic cycle with varying latent periods and infectious periods from 3 to 25 days. We observed multi-annual cycles when both the latent and infectious periods were sufficiently short (white regions in Fig. 2c). The length of the epidemic cycle is close to the length of the cycle of the fluctuating transmission rate (an annual cycle) when the latent or infectious periods are large, as observed in *MP* epidemics (latent period = 2 to 3 weeks and infectious period = 2 to 3 weeks for *MP*) (Fig. 2c), thus school-term forcing does not explain the periodicity of *MP* (i.e. four years), which is much larger than the school term cycle (i.e. one year). Regarding the amplitude of school-term forcing, we explored the epidemic cycle when the strength of the school-term forcing, *b*, was varied from 1.1 to 17 (this range includes realistic *b* values, as the estimated *b* for measles in the U.K. is 1.7^27^), however, school-term forcing always produces annual cycles with *MP* parameter settings, i.e., latent period = 2 to 3 weeks and infectious period = 2 to 3 weeks (Fig. 2).

### Epidemiological interference

Epidemiological interference between strains by cross-immune protection is known to change the periodicity of epidemics^25^. A combination of cross-immune protection and school-term forcing may explain the *MP* epidemic cycle. We also examined whether the presence of both seasonally-forced transmission rates and cross-immune protection can capture the *MP* epidemic cycle. By varying the strength of cross-immune protection (with a range from 0 to 1), mean duration of immunity (with the range from two to ten years), and the ratio of transmission rates from school days to holidays, *b*, (with a range from 0.1 to 20), only annual cycle oscillation was observed. Even when both cross-immune reaction and seasonally-forced transmission rates were taken into account, the four-year epidemic cycle of *MP* was not explained, nor was it observed within our model.

### Epidemic periodicity when the sojourn time of each infectious state follows a non-exponential distribution

#### Without cross-immune protection

We investigated the contribution of minor variance in three sojourn times, i.e. the latent period, the infectious period, and the duration of immunity. For the sake of simplicity, we assumed variance of the sojourn time is either zero (delta) or mean-squared (exponential). Minor variance in the duration of immunity is critical for the oscillation of epidemics (Table 2). On the other hand, variance in the length of the latent and infectious periods is not important for oscillation. Furthermore, the mean of the length of the latent and infectious periods is also not sensitive to the oscillation in long-term *MP* transmission dynamics (please see Supplemental Information Figure S1a,b for the details). This analysis was conducted using a deterministic model.

To analyze the detailed effect of minor variance in the duration of immunity, we also used a stochastic model to investigate the relationship between the oscillation of the epidemic and non-zero, but small, variance in the duration of immunity. The strength of oscillation of the epidemic curve is measured by the coefficient of variation, CV (see Methods). Weak oscillation shows small CV and strong oscillation shows large CV (top panels in Fig. 3c). Our simulation results show the smaller the variance in the duration of immunity, the larger the CV (bottom panel in Fig. 3c). Threshold-like behavior in the variance of the duration of immunity for the oscillation of epidemics was also observed. Since CV for the *MP* epidemic in Japan from 1982 to 1990 is 0.7^3^, the intermediate variance in the duration of immunity between zero (constant immunity duration) and the square of its mean (immunity duration following an exponential distribution) can explain *MP* epidemic dynamics.

Not only sustained oscillation in *MP* epidemics, but also multi-annual epidemic cycles not explained by school-term forcing, are observed with minor variance in the duration of immunity (Fig. 3b). The epidemic cycle depends on both the mean and the variance in the duration of immunity.

#### With cross-immune protection

Even if *MP* serotypes interfere with each other by cross-immune protection, the periodicity of epidemics is determined by variance of the duration of immunity as similar to a stochastic model which disregards cross-immunity; the smaller the variance, the stronger the epidemic oscillation (Fig. 4). Various epidemic cycles, including three to five year cycles observed in Denmark, the U.S., and Japan, were observed. The effect of the mean duration of immunity and the strength of cross-immune protection on the epidemic cycle are less clear than the variance of the immunity period (Fig. 5).

## Discussion

In this study, we assessed which assumptions in theoretical studies of childhood diseases are required to explain *MP* epidemic periodicity. Our results show that minor variation in the duration of immunity is essential to capturing *MP* epidemic periodicity, and also suggest that ordinary differential equations, ODE, are not suitable for describing *MP* epidemics. Oscillation of the epidemic curve is robust even if there is epidemiological interference due to cross-immune protection, which is observed as negative correlation between epidemics per *MP* serotype.

**Figure 1:**
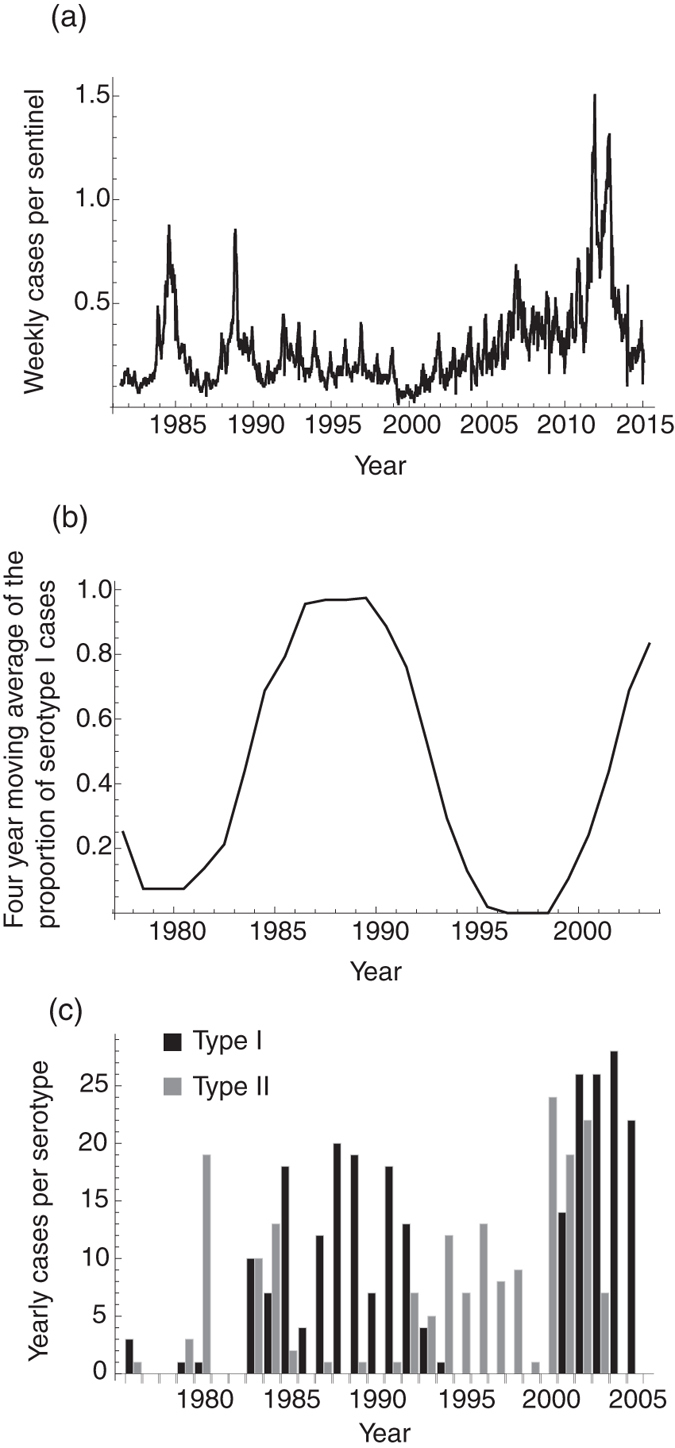
*MP* incidence in Japan. (**a**) Time series data of weekly *MP* cases per sentinel in Japan from 1981 to 2015. The figure shows the number of reported primary atypical pneumonia cases until March 1999 and *MP* cases after April 1999 due to the change in the law for reporting infectious diseases. The data were collected from nationwide sentinel clinics and integrated by the Infectious Diseases Surveillance Center, National Institute of Infectious Diseases, Japan (http://idsc.nih.go.jp/index.html). (**b**) Annual detection rate of serotype I among all reported *MP* cases with the four-year moving average. (**c**) Time series data of yearly cases per serotype from 1976 to 2005. The data shown in (**a**) were collected from the whole area of Japan while most data shown in (**b**,**c**) were collected from Kanagawa prefecture in Japan.

**Figure 2:**
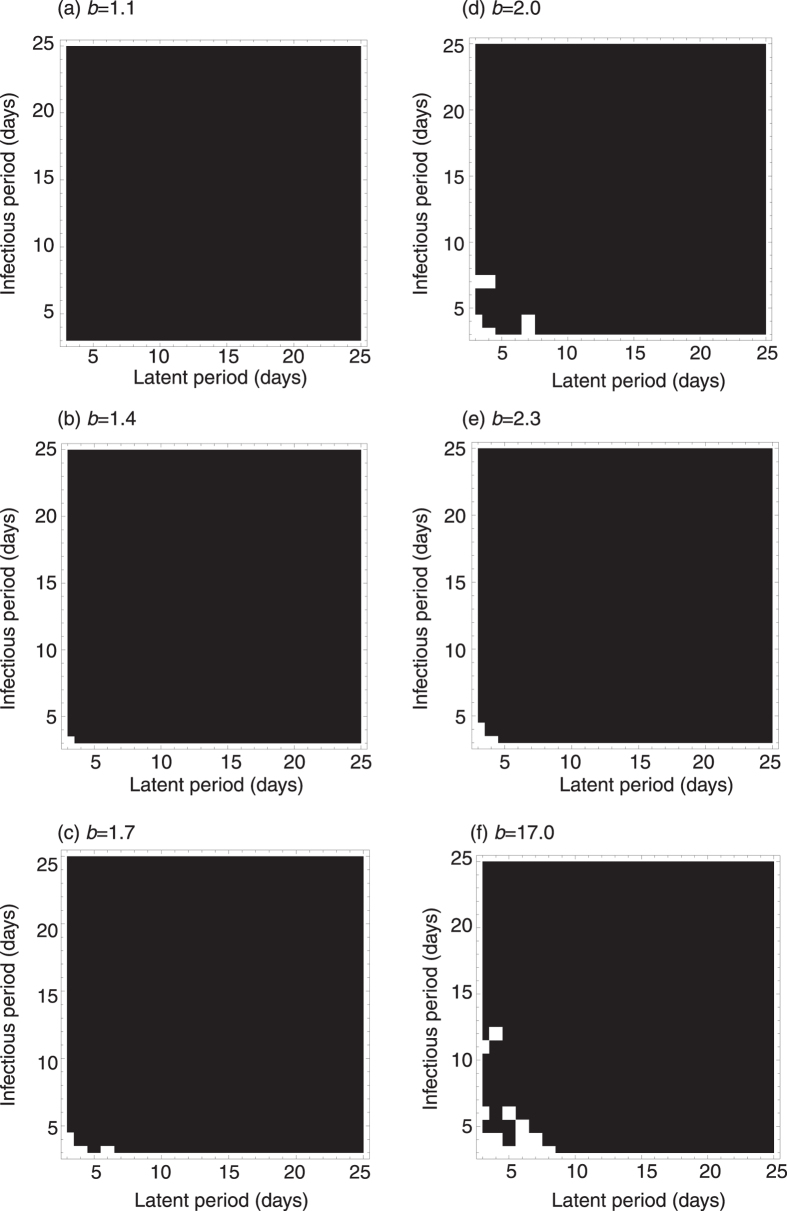
Epidemic cycles with varied infectious periods and latent periods. Short latent and infectious periods are required for epidemic cycles longer than one year (the periodicity of school terms). The latent and infectious periods for *MP* are both 2 to 3 weeks. Black denotes annual epidemic cycles and white denotes epidemic cycles longer than one year. To explore the possibility of oscillation the epidemic cycles were calculated with varied *b* from 1.1 to 17.0 (the estimated *b* for measles cases in the U.K. is 1.7^27^). Mean duration of immunity is assumed to be 7 years.

**Figure 3:**
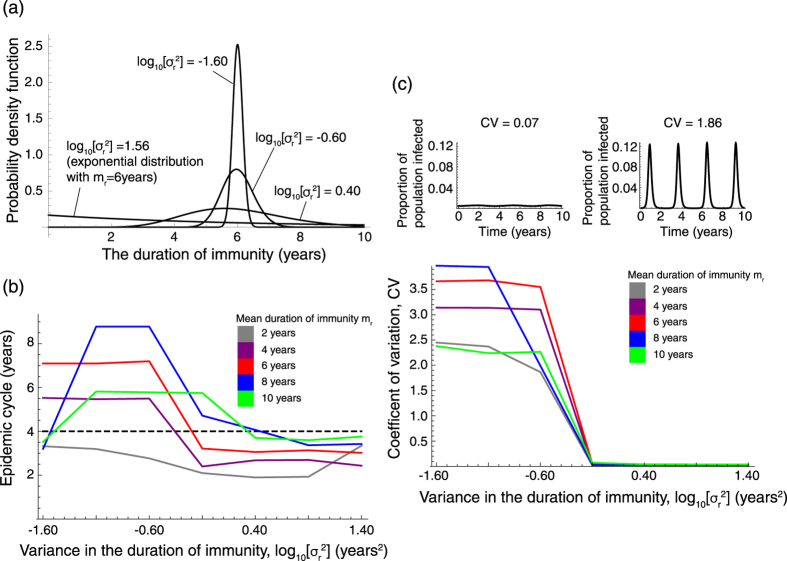
The impact of variance in the duration of immunity on *MP* epidemics. (**a**) The distribution of the immunity duration with mean = 6 years and varied variance. We assumed Δ*t* = 1/200 years. (**b**) The relationship between variance in the duration of immunity and the epidemic cycle. The dashed line shows the 4 year cycle observed in Japan. (**c**) Variance in the duration of immunity determines the oscillation of the epidemic curve.

**Figure 4:**
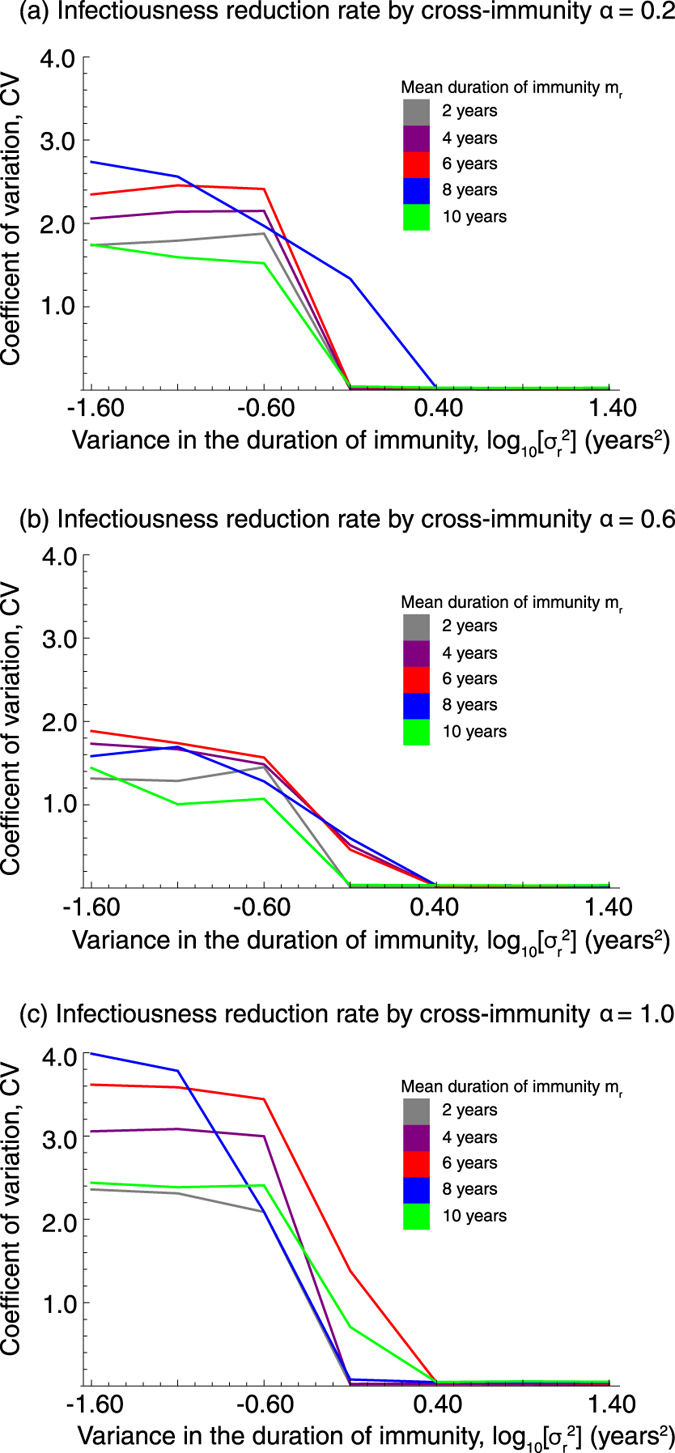
The impact of epidemiological interference by cross-immunity on the oscillation of the epidemic curve. The strength of the cross-immune reaction is denoted by *α*; *α* = 0 means no cross-immune reaction and *α* = 1 means perfect protection by cross-immune reaction. The coefficient of variation, CV, of the number of people infected with any strain was measured. CV is a measure of the variation in the number of infected individuals over time.

**Figure 5:**
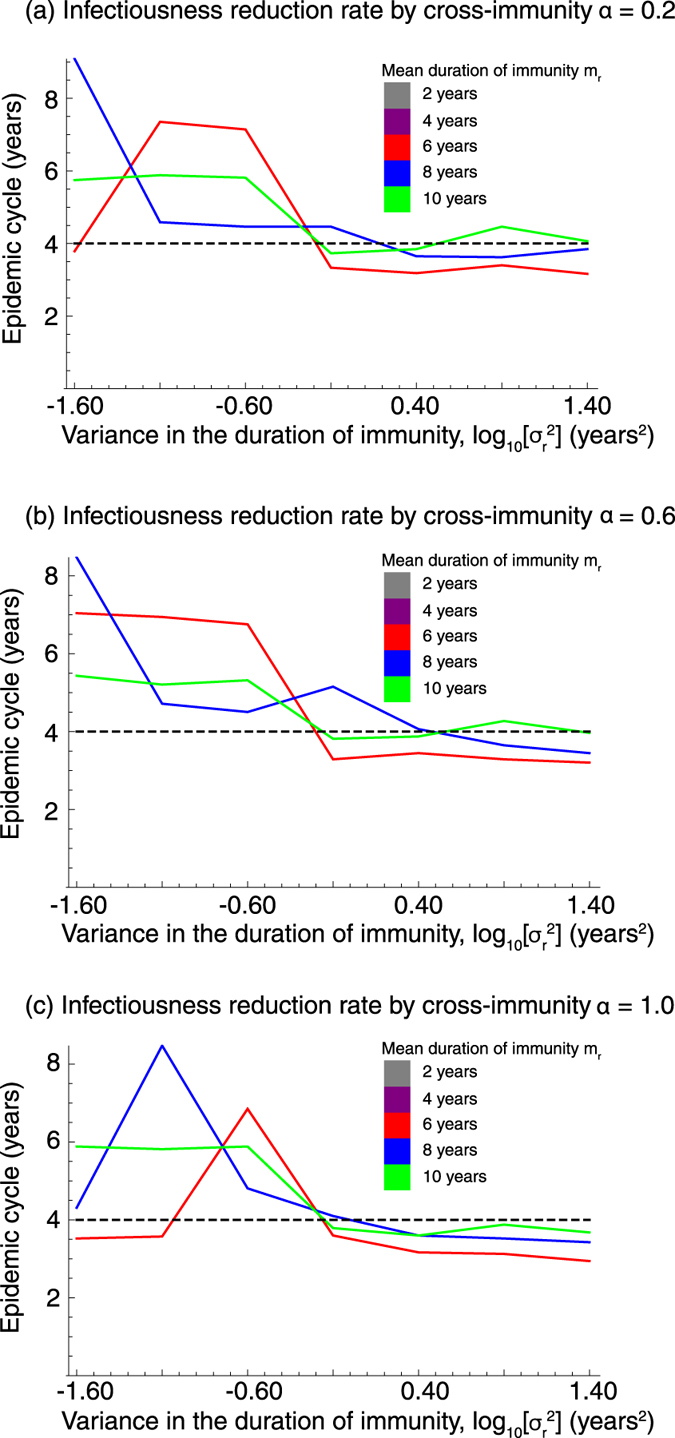
The impact of epidemiological interference on the *MP* epidemic cycle. The epidemic cycle for the number of people infected with any strain was measured. The dashed line shows the 4-year cycle observed in Japan.

**Figure 6:**
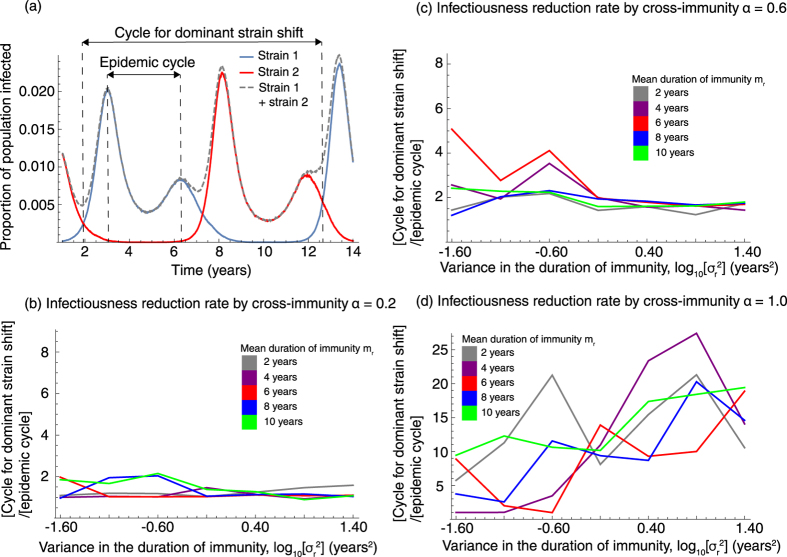
The impact of epidemiological interference on the cycle for dominant strain shift. (**a**) Illustrates the relationship between the cycle for dominant shift and the epidemic cycle on the *MP* epidemic with *m*_r_ = 8 years, log_10_[*σ*_r_^2^] = −0.60 and *α* = 0.6. (**b**–**d**) show the cycles for dominant strain shift with varied strength of cross-immune protection *α*.

Both the epidemic cycle and the shape of the epidemic curve, i.e. the coefficient of variation, are less sensitive to the strength of cross-immune reactions. At the same time, the cycle of dominant strain shift is sensitive to cross-immunity (Fig. 6b–d). Previous studies show that stronger cross-immunity causes longer periodicity for dominant strain shifting^12^. *MP* epidemics in Kanagawa prefecture, Japan in 1976–1991 show 16 to 20 year cycles for dominant strain shift (Fig. 1b)^3^. Long periodicity for dominant strain shift, which takes the duration of four to five *MP* epidemic cycles, suggests strong cross-immunity between strains. Indeed, our results showed similar tendency for the relationship between strength of cross-immunity and the cycle of dominant strain shift. Short timespan dominant strain shifts (with duration of one to two epidemic cycles) are observed if cross-immunity is weak (Fig. 6b–d). This result suggests that the strength of cross-immunity can be estimated from the time between switching dominant strains.

A previous study showed positive correlation between the mean duration of immunity and epidemic cycles^21^. However, this tendency was not clearly visible in our results. This lack of a clear relationship observed in our results may be caused by an occurrence of switching between attractors. Previous theoretical studies show multiple solutions for epidemic cycles with a fixed mean duration of immunity even if there is no epidemiological interference from cross-immune reactions^21^. Our simulations also confirmed these results of multiple epidemic cycles as shown in Figures S2. This warrants future work on the possible existence of conditions of multiple attractors and transitions between them.

To predict *MP* prevalence per serotype, the estimation of the immunity duration distribution is essential. However, degeneracy of the epidemic cycle, i.e. the existence of multiple attractors, suggests the difficulty involved in the estimation of the mean duration of immunity solely from prevalence time-series data. In addition to multiple attractors, transmission dynamics are complicated by epidemiological interference between serotypes. The prevalence for each serotype is necessary to fully capture the dynamics, however, serotype information was not reported except for a few studies, as shown in Fig. 1c and Nguipdop-Djomo *et al.* 2013^28^. A possible strategy for estimating the mean duration of immunity would be to use the age-specific sero-prevalence for each serotype. The simplest catalytic model frequently used for epidemiological parameter estimation from serological data is not applicable for *MP* because of the periodicity of the epidemics. Extrapolation of time-series data of prevalence may enable us to estimate both the mean and the variance of the duration of immunity.

In this study we did not take into account the realistic age structure of the host population, while a previous theoretical study showed that age structure may explain sustained oscillation of the epidemic curve^24^. Age structure is unlikely to be essential for *MP* epidemic periodicity, because disappearances of oscillation in *MP* prevalence were observed at similar timing in different countries. Synchronization of similar changes of age structure in different countries at similar timing is required to explain the disappearance of the *MP* epidemic periodicity, however this is unrealistic. Disappearances of oscillation in *MP* incidence may be explained better by the change in the distribution for the duration of immunity due to widespread use of macrolide antibiotics in those countries.

Throughout this study we assumed a constant fraction of infections become symptomatic over time. *R*_0_ from Nguipdop-Djomo *et al.* 2013^28^ was used in our analysis as the baseline scenario and is estimated from sero-prevalence. Our results for *MP* prevalence use the sum of symptomatic and asymptomatic cases. If the proportion of infections that become symptomatic is the same over time, *MP* periodicity is independent from the symptomatic case rate.

In summary, minor variation in the duration of immunity at the population level is the most likely determinant of the periodicity of *MP* epidemics. If the distributions of latent, infection, and immunity periods follow exponential distribution, epidemic cycles longer than one year were not reproducible, even if both seasonal forcing of transmission (school-term forcing) and cross-immune protection were considered as the driving forces of multi-annual epidemic cycles^11^,^15^,^16^,^25^,^29^. In short, only non-exponentially distributed variation in the duration of immunity produced multi-annual epidemic cycles. The cyclic incidence pattern induced by minor variation in the duration of immunity is robust even if there is epidemiological interference due to cross-immune protection. Since small variance in the duration of immunity can induce complex dynamics, more detailed analysis is required to understand *MP* epidemic dynamics; even though the analysis of the compartmental model with non-exponential distributed sojourn time is an analytically challenging issue.

## Methods

From *MP* clinical observations, symptoms appear two to three weeks after infection, and continue for an additional two to three weeks^30^. The latent period may be different from the incubation period, but as the latent period is not known, we assumed the latent period to be the same duration as the incubation period (*m*_e_ = 3 weeks). Based on the natural history of *MP*, the whole population can be classified by infection state: susceptible *s*, exposed *e*, infectious *i*, recovered *r*. Re-infection with *Mycoplasma pneumoniae* is frequently observed, suggesting waning acquired immunity, so we employed the SEIRS model.

The age distribution of *MP* infection peaks in children and young adults^14^. For the sake of simplicity, we focused only on the prevalence among the populations of children and young adults. We also assumed a constant host population; the rate of birth and outflow of host by death or aging are the same, *μ*. As a result, we focused on a 15 year range for the high risk age group (1/*μ* = 15 years).

Compartmental models in epidemiology, such as the SIR model, are described by ordinary differential equations which assume the sojourn time in each compartment follows an exponential distribution. To relax this strong assumption, we assume that the sojourn time in infection state *x*, *τ*_x_, follows a gamma distribution *G*_x_,


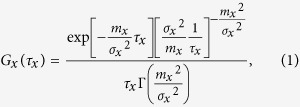


where *m*_x_ and *σ*_x_^2^ are the mean and variance of *τ*_x_, and Γ is the gamma function. If *σ*_x_^2^ = *m*_x_^2^, the distribution of *τ*_x_ is equal to the exponential distribution and the system is equivalent to a system described by ordinary differential equations. If *σ*_x_^2^ = 0, the distribution of *τ*_x_ is equal to the delta distribution and the system is equivalent to a system described by delay differential equations with a fixed delay. To analyze the impact of *σ*_x_^2^ on the dynamics of *MP* epidemics, we explored the system with varying *σ*_e_^2^, *σ*_i_^2^, and *σ*_r_^2^ with ranges from 0 to *m*_e_^2^, *m*_i_^2^, and *m*_r_^2^, respectively. When *τ*_e_, *τ*_i_, and *τ*_r_ follow an exponential distribution (*σ*_e_^2^ = *m*_e_^2^, *σ*_i_^2^ = *m*_i_^2^, *σ*_r_^2^ = *m*_r_^2^), the time series change of each compartment is described as follows:


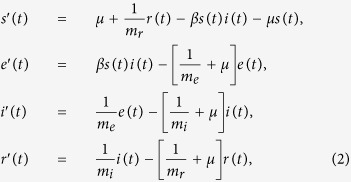


where each prime denotes a time derivative. Each variable represents the population fraction so that *S*(*t*) + *e*(*t*) + *i*(*t*) + *r*(*t*) = 1 holds for *t* > 0. When *τ*_e_ and *τ*_i_ follow exponential distributions (*σ*_e_^2^ = *m*_e_^2^, *σ*_i_^2^ = *m*_i_^2^) and *τ*_r_ follows a gamma distribution, the time series change of each compartment is described as follows:


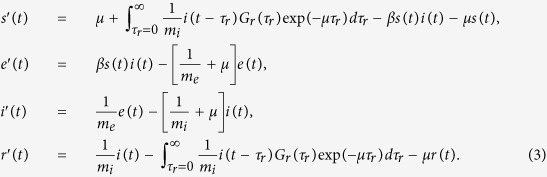


The integral term in equation (3) describes the proportion of those who lose immunity per unit time at time *t*; namely, the transition from compartment *r* to compartment *s* at time *t*. When the sojourn time follows a non-exponential distribution, the transition rate to the next compartment depends on the sojourn time at the current compartment. To calculate the transition to the next compartment (compartment *s*), it is necessary to calculate the transition of individuals per elapsed sojourn time at the current compartment (compartment *r*). The proportion of individuals belonging to compartment *r* with elapsed sojourn time at compartment *r*, *τ*_r_, is given by the product of the initial proportion of transition from compartment *i*, 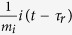, the transition probability per unit time to compartment *s* at sojourn time *τ*_r_, *G*_*r*_(*τ*_*r*_), and the survival probability at the sojourn time *τ*_r_, exp(−*μτ*_*r*_). The parameter values used in this paper are summarized in Table 1. The transmission rate, *β*, is derived by solving *R*_0_ = 1.7 for each parameter set. The basic reproduction number, *R*_0_, is given by


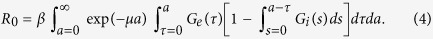


*R*_0_ of the SEIRS model is defined by the expected number of secondary cases from a single exposed individual in a fully susceptible population^31^, and can be decomposed to the product of the transmission rate *β*, the survival probability of initially exposed individuals exp(−*μa*), and the probability that an initially exposed individual has infectiousness after *a* unit time elapsed since the infection (here *τ* can be interpreted as the elapsed time since the infection when the initially exposed individual leaves compartment *e* and enters compartment *i*), i.e., belongs to compartment *i*, 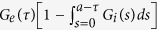. For the analysis utilizing the assumption that the sojourn time follows an intermediate distribution between delta and exponential distributions, i.e., 0 < *σ*_x_^2^ < *m*_x_^2^, we employed individual-based Monte Carlo simulation (IBM), the details of which are shown in the “*Cross-immunity*” section under Methods.

### School-term forcing transmission

The school year in Japan is divided into three terms: the first trimester is from 7 April to 18 July, the second trimester is from 1 September to 22 December, and the third trimester is from 7 January to 24 March for 2014. The length of semesters is similar between different years. As with previous mathematical models for school-term forcing^26^, we assumed transmission rates during semesters, *β*_school days_, are constant over time and higher than that on other days, *β*_holidays_. To determine *β*_school days_ and *β*_holidays_, we introduce a parameter, *b*, describing the ratio of transmission rate between school days and holidays (i.e., *b* = *β*_school days_/*β*_holidays_), and a parameter, *β*, describing the annual average of *β*_school days_ and *β*_holidays_ (i.e., *β* = ([number of holidays in a year]*β*_*holidays*_ + [number of school days in a year]*β*_*school days*_)/[length of a year]). Using *β* and *b*, *β*_school days_ and *β*_holidays_ are given by


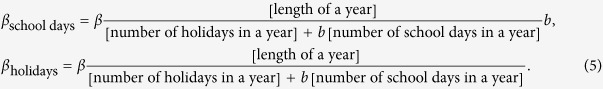


### Cross-immunity

To clarify the impact of cross-immunity on the epidemic dynamics of *MP* we expand the above model to incorporate a reduction of susceptibility due to previous *MP* infection. We assumed that acquired immunity from the infection with a strain reduces susceptibility to other strains, and this immune cross-protection decays with waning acquired immunity. When the prevalence of multiple strains interferes with one another via cross-immune protection, compartments for all combinations of infection states against all strains are required to capture the epidemic dynamics using deterministic models. For example, there are two serotypes and four infection states for each serotype (*s*, *e*, *i*, and *r*), therefore 4^2^, or 16, compartments are required. Furthermore, when the sojourn time in each compartment does not follow an exponential distribution, many more compartments are required and the model structure becomes much more complicated. To avoid complexity, we constructed an IBM describing co-circulation of two serotypes.

We considered a host population of two million and tracked the timing of transition between the infection states. The sojourn time in each infection state is determined using a gamma random number generator with the parameters *σ*_*x*_^2^ and *m*_*x*_^2^. Transition of the infection state occurs after the lapse of determined sojourn time. Transmission probability is determined by the current infection state of each individual. If the individual does not have immunity to any strain, the transmission probability of strain *x* per unit time *Δt* is


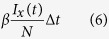


where *I*_*x*_ is the number of infected individuals with strain *x* and *N* is total number of hosts. In this study we set *Δt* = 1/200 year (≈1.8 days). The transmission probability of a strain for an individual is reduced by *α* if he has immunity against another strain. For the analysis of a model disregarding cross-immunity with arbitral *σ*_*x*_^2^ having a range of 0 < *σ*_x_^2^ < *m*_x_^2^, IBM was used with *α* = 0. Birth, death, and outflow of hosts from the population by aging occur randomly with a given rate, *μ*.

### Analysis of IBM results

In all analyses the IBM results during the first 200 years were discarded and the number of infected individuals during the next 500 years were recorded. The epidemic cycle and the cycle of dominant strain shift was measured by power spectral analysis with Fourier transformation. The epidemic cycle, in the presence of epidemiological interference by cross-immune protection, was measured based on field data regarding the number of people infected with any *MP* serotype. The strength of oscillation of the epidemic curve was measured by the coefficient of variation (CV) for the number of infected individuals over time, CV = [standard deviation]/[mean].

## Additional Information

**How to cite this article**: Omori, R. *et al.* The determinant of periodicity in *Mycoplasma pneumoniae* incidence: an insight from mathematical modelling. *Sci. Rep.* **5**, 14473; doi: 10.1038/srep14473 (2015).

## Supplementary Material

Supplementary Information

## Figures and Tables

**Table 1:** The parameter values used in our simulations.

Symbol	Description	Baseline value	Reference
*R*_0_	Basic reproductive number	1.7	28
*m*_e_	Mean latent period	21 days	30
*m*_i_	Mean infectious period	21 days	30
*m*_r_	Mean duration of immunity	7 years (2–10 years)	32
*σ*_e_^2^	Variance of latent period	*m*_e_^2^	Assumed
*σ*_i_^2^	Variance of infectious period	*m*_i_^2^	Assumed
*σ*_r_^2^	Variance of immunity duration	Varied	Assumed
*α*	Infectiousness reduction rate by cross-immune protection	Varied	Assumed
1/*μ*	High risk age group duration	15 years	Assumed

**Table 2:** The distribution of the sojourn time in class *r* determines the oscillation of *MP* epidemics.

	Distribution of sojourn time in the class *e*	Distribution of sojourn time in the class *i*	Distribution of sojourn time in the class *r*	Oscillation
Scenario 1	Exponential	Exponential	Exponential	No oscillation
Scenario 2	Delta	Exponential	Exponential	No oscillation
Scenario 3	Exponential	Delta	Exponential	No oscillation
Scenario 4	Delta	Delta	Exponential	No oscillation
Scenario 5	Exponential	Exponential	Delta	Oscillate
Scenario 6	Delta	Exponential	Delta	Oscillate
Scenario 7	Exponential	Delta	Delta	Oscillate
Scenario 8	Delta	Delta	Delta	Oscillate

“Exponential” denotes exponential distribution and “Delta” denotes delta distribution.
