# Supplementary material for: The determinant of periodicity in Mycoplasma pneumoniae incidence: an insight from mathematical modelling
Source: Sci Rep. 2015 Sep 28;5:14473. doi: 10.1038/srep14473 (PMC4585982; doi:10.1038/srep14473)
Supplement: Supplementary Information [file srep14473-s1.pdf]

Supplementary information

The determinant of periodicity in *Mycoplasma pneumoniae* incidence: an insight from mathematical modelling

Ryosuke Omori[1], Yukihiko Nakata[2], Heidi L. Tessmer[1], Satowa Suzuki[3], and Keigo Shibayama[3]

[1]*Division of Bioinformatics, Research Center for Zoonosis Control, Hokkaido University, Sapporo, Hokkaido, Japan*

[2]*Graduate School of Mathematical Sciences, The University of Tokyo, 3-8-1 Komaba Meguro-ku, Tokyo 153-8914, Japan*

[3] *Department of Bacteriology II, National Institute of Infectious Diseases, Tokyo, Japan*

Supplementary figures

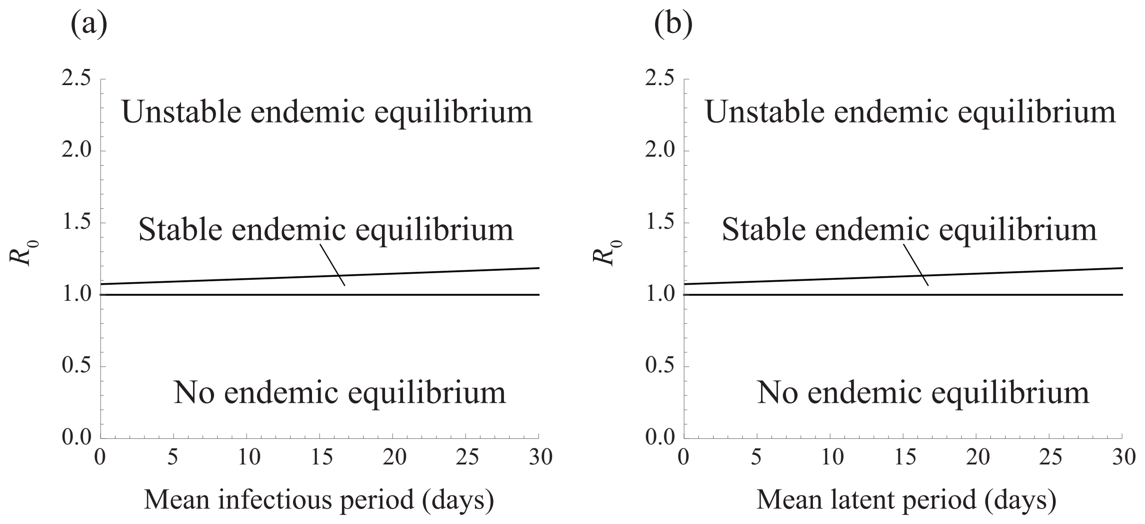

Figure S1: Oscillation in the long-term transmission dynamics of *MP* is not significantly sensitive to the length of the infectious period (a) or to the length of the latent period (b) if the basic reproduction number does not vary. Parameters are fixed as $m_r = 4$ (years) in both figures and $m_e = 21$ (days) in (a), while $m_i = 21$ (days) in (b). The bottom line shows the boundary for the existence of endemic equilibrium and the upper line shows the boundary for the stability of endemic equilibrium.

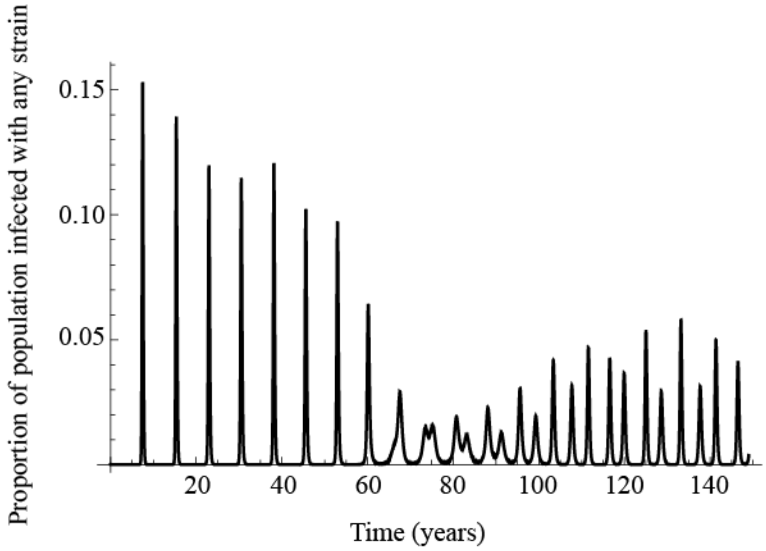

Figure S2: Change in epidemic cycle over time. No epidemiological interference was assumed and the parameters are set as m_r = 6 years, $\log_{10}[\sigma_r^2]$ = -0.60.

Stability analysis of an SEIRS model

Here we derive parametric expressions that are respectively plotted in Figures S1 (a) and (b) to determine stability boundaries in two-parameter planes, assuming a delta distribution for the waning immunity, i.e. $G_r(\tau_r) = \delta_{m_r}(\tau_r)$.

Since the demographic process is slow compared to the disease transmission dynamics, here we may assume $\mu = 0$ to facilitate the mathematical analysis. The model (3) in the main text is written as

$$\begin{aligned} s'(t) &= -\beta s(t)i(t) + \frac{1}{m_i} i(t - m_r), \\ e'(t) &= \beta s(t)i(t) - \frac{1}{m_e} e(t), \\ i'(t) &= \frac{1}{m_e} e(t) - \frac{1}{m_i} i(t), \\ r'(t) &= \frac{1}{m_i} i(t) - \frac{1}{m_i} i(t - m_r). \end{aligned} \tag{S1}$$

First, let us compute the endemic equilibrium for (S1). The endemic equilibrium is denoted by $(\bar{s}, \bar{e}, \bar{i}, \bar{r})$. Then one can find that

$$\bar{s} = \frac{1}{\beta m_i}$$

and equalities $\bar{e} = \frac{m_e}{m_i}\bar{i}$ and $\bar{r} = \frac{m_r}{m_i}\bar{i}$. Since $\bar{s} + \bar{e} + \bar{i} + \bar{r} = 1$ holds (the total fraction is one), we get

$$\bar{i} = \frac{1 - \frac{1}{\beta m_i}}{1 + \frac{m_e}{m_i} + \frac{m_r}{m_i}}.$$

Linearizing (S1) around the equilibrium, we can derive the characteristic equation, see e.g. [1] for detail. Let I be the 3×3 identity matrix. The characteristic equation is

$$\det\left(\begin{pmatrix} -\beta\bar{i} & 0 & m_i^{-1}e^{-\lambda m_r} - m_i^{-1} \\ \beta\bar{i} & -m_e^{-1} & m_i^{-1} \\ 0 & m_e^{-1} & -m_i^{-1} \end{pmatrix} - \lambda I\right) = 0. \tag{S2}$$

Below we use the notation i to denote the imaginary unit i.e. $i = \sqrt{-1}$, thus we write Λ for $\beta\bar{i}$. The equation (S2) is

$$\begin{aligned}\lambda^3 + (\Lambda + m_e^{-1} + m_i^{-1})\lambda^2 + \Lambda(m_e^{-1} + m_i^{-1})\lambda + \Lambda m_e^{-1}m_i^{-1} \\ = \Lambda m_e^{-1}m_i^{-1}e^{-\lambda m_r}.\end{aligned} \tag{S3}$$

We now investigate a parameter set such that (S3) has purely imaginary roots to see if the endemic equilibrium becomes unstable via Hopf bifurcation. Substituting $\lambda = i\omega,\ \omega > 0$ into (S3) we get the following two equations

$$-(\Lambda + m_e^{-1} + m_i^{-1})\omega^2 + \Lambda m_e^{-1}m_i^{-1} = \Lambda m_e^{-1}m_i^{-1}\cos(\omega m_r), \tag{S4a}$$

$$-\omega^3 + \Lambda(m_e^{-1} + m_i^{-1})\omega = -\Lambda m_e^{-1}m_i^{-1}\sin(\omega m_r). \tag{S4b}$$

From the first equation (S4a) we get

$$\Lambda = \frac{(m_e^{-1} + m_i^{-1})\omega^2}{m_e^{-1}m_i^{-1}\{1 - \cos(\omega m_r)\} - \omega^2}. \tag{S5}$$

Plugging (S5) into the second equation (S4b) we get

$$\begin{aligned}0 &= \{m_e^{-1}\sin(\omega m_r) + \omega\}m_i^{-2} \\ &+ m_e^{-1}[\omega\{1 + \cos(\omega m_r)\} + m_e^{-1}\sin(\omega m_r)]m_i^{-1} + \omega(\omega^2 + m_e^{-2}).\end{aligned} \tag{S6}$$

From (S6) one has

$$\begin{aligned}0 &= \omega(\omega^2 + m_e^{-2})m_i^2 + \\ &m_e^{-1}[\omega\{1 + \cos(\omega m_r)\} + m_e^{-1}\sin(\omega m_r)]m_i + \{m_e^{-1}\sin(\omega m_r) + \omega\}.\end{aligned} \tag{S7}$$

One can solve (S7) with respect to m_i as

$$m_i = m_i^*(\omega) := \frac{-m_e^{-1}[\omega\{1+\cos(\omega m_r)\} + m_e^{-1}\sin(\omega m_r)] \pm \sqrt{D_e(\omega)}}{2\omega(\omega^2 + m_e^{-2})}, \tag{S8}$$

where

$$\begin{aligned} D_e(\omega) := \quad & m_e^{-2}[\omega\{1+\cos(\omega m_r)\} + m_e^{-1}\sin(\omega m_r)]^2 \\ & -4\{m_e^{-1}\sin(\omega m_r) + \omega\}\omega(\omega^2 + m_e^{-2}). \end{aligned}$$

If we plug (S8) into (S5) we get

$$\Lambda = \Lambda_e^*(\omega) := \frac{\{m_e^{-1} + m_i^*(\omega)^{-1}\}\omega^2}{m_e^{-1} m_i^*(\omega)^{-1}\{1-\cos(\omega m_r)\} - \omega^2}.$$

Finally using the relation

$$\Lambda = \frac{\beta m_i - 1}{m_i + m_e + m_r} = \frac{R_0 - 1}{m_i + m_e + m_r},$$

we get a parametric expression for R_0 as

$$R_0 = 1 + \Lambda_e^*(\omega)\{m_i^*(\omega) + m_e + m_r\}. \tag{S9}$$

Fixing m_e and m_r, we use expressions (S8) and (S9), in the (m_i, R_0) parameter plane, to plot the parameter set, where the characteristic equation (S3) has purely imaginary roots (Figure S1 (a)). We numerically checked stability of the endemic equilibrium as depicted in Figure S1 (a). Indeed if two parameters (m_i, R_0) cross the plotted line from below to above, Hopf bifurcation is expected as purely imaginary roots $\pm i\omega$ move to the right half complex plane. Elaboration of the analysis will be presented elsewhere.

To work in the (m_e, R_0)-parameter plane, we can repeat the same procedure, fixing m_i and m_r. Since (S6) is a symmetric polynomial for m_e and m_i, one can solve (S6) as

$$m_e = m_e^*(\omega) := \frac{-m_i^{-1}[\omega\{1+\cos(\omega m_r)\} + m_i^{-1}\sin(\omega m_r)] \pm \sqrt{D_i(\omega)}}{2\omega(\omega^2 + m_i^{-2})}, \tag{S10}$$

where

$$D_i(\omega) := \quad m_i^{-2}\{\omega\{1+\cos(\omega m_r)\} + m_i^{-1}\sin(\omega m_r)\}^2$$
$$-4\{m_i^{-1}\sin(\omega m_r) + \omega\}\omega(\omega^2 + m_i^{-2}).$$

Then from (S5) one obtains

$$\Lambda = \Lambda_i^*(\omega) := \frac{\{m_e^*(\omega)^{-1} + m_i^{-1}\}\omega^2}{m_e^*(\omega)^{-1} m_i^{-1}\{1-\cos(\omega m_r)\} - \omega^2}.$$

We thus get a parametric expression for R_0 as

$$R_0 = 1 + \Lambda_i^*(\omega)\{m_i^*(\omega) + m_e + m_r\}. \qquad \text{(S11)}$$

Similarly we can plot a parametric curve given by (S10) and (S11) in the (m_e, R_0)-parameter plane, see Figure S1 (b).

Reference

1. O. Diekmann, S.A. van Gils, S.M.V. Lunel, H.O. Walther, Delay Equations Functional, Complex and Nonlinear Analysis, Springer Verlag (1991).
